# On the Performance of Decode-and-Forward Equal-Gain-Combining Relay Systems over Weibull Fading Channels

**DOI:** 10.3390/s23063174

**Published:** 2023-03-16

**Authors:** Paula Tilleria Lucero, Henry Carvajal Mora, Nathaly Orozco Garzón, Fernando Almeida García

**Affiliations:** 1 Faculty of Engineering and Applied Sciences (FICA), Telecommunications Engineering, Universidad de Las Américas (UDLA), Quito 170503, Ecuador; 2 School of Electrical and Computer Engineering, University of Campinas (UNICAMP), Campinas 13083-852, Brazil

**Keywords:** decode-and-forward, equal-gain-combining, Weibull fading, outage probability, average bit error probability

## Abstract

Relay-assisted wireless communications, where both the relay and the final destiny employ diversity-combining techniques, represent a compelling strategy for improving the signal-to-noise ratio (SNR) for mobile terminals, mainly at millimeter-wave (mmWave) frequency bands. In this sense, this work considers a wireless network that employs a dual-hop decode-and-forward (DF) relaying protocol, in which the receivers at the relay and at the base station (BS) use an antenna array. Moreover, it is considered that the received signals are combined at reception using equal-gain-combining (EGC). Recent works have enthusiastically employed the Weibull distribution so as to emulate the small-scale fading behavior in mmWave frequencies, which also motivates its use in the present work. For this scenario, exact and asymptotic expressions for the system’s outage probability (OP) and average bit error probability (ABEP) are derived in closed form. Useful insights are gained from these expressions. More precisely, they illustrate how the system and fading parameters affect the performance of the DF-EGC system. Monte Carlo simulations corroborate the accuracy and validity of the derived expressions. Furthermore, the mean achievable rate of the considered system is also evaluated via simulations. Useful insights regarding the system performance are obtained from these numerical results.

## 1. Introduction

Wireless communication systems are affected by diverse phenomena, among them, multipath fading produces a random attenuation over the received signals. This phenomenon can be emulated using different statistical distributions depending on the operating scenario. For instance, the Rayleigh and Rice distributions are typically considered for non-line-of-sight (NLOS) and line-of-sight (LOS) propagation scenarios, respectively [1]. However, these distributions do not adequately emulate the fading behavior at millimeter-wave (mmWave) frequencies. As a consequence, other more general distributions, such as the Weibull distribution, have been considered for these scenarios [2,3,4]. In addition, it has been shown that the Weibull distribution is a versatile fading model due to its flexibility and excellent fit to experimental data [5,6]. Moreover, the Weibull fading model encompasses, as special cases, the Rayleigh, exponential and one-sided Gaussian distributions. Interestingly, the authors in [7] have shown that, unlike other distributions, the Weibull fading model allows emulating propagation scenarios worse (more severe) than those generated by Rayleigh fading, mainly for vehicle-to-vehicle (V2V) communications and wireless sensor network (WSN) scenarios. These applications are encompassed within the ultra-reliable low-latency communications (URLLC) and the massive machine-type communications (mMTC) use cases, respectively, which are established for fifth-generation (5G) and beyond 5G (B5G) mobile networks [8,9].

Several works have focused on enhancing the coverage and performance of wireless systems by counteracting the detrimental effects of fading. Diversity and combining techniques have been extensively studied in the literature [10,11,12,13,14]. Such techniques focus on processing various replicas of the transmitted signal, which are affected by independent fading [15,16]. The analysis of diversity-combining techniques involves the study of the statistics of the sum of random variables [17]. Thus, the probability density function (PDF) and the cumulative distribution function (CDF) of the sum of random variables are fundamental for evaluating the performance of wireless systems employing diversity techniques. In [18], the authors found an expression to approximate the PDF of the sum of independent and identically distributed (i.i.d.) Weibull random variables. More recently, exact expressions for the PDF and the CDF of the sum of i.i.d. Weibull random variables were derived in [19].

Among the different combination techniques proposed, maximal-ratio-combining (MRC) and equal-gain-combining (EGC) stand out [20,21,22]. In particular, MRC is the best combination technique, but it requires the estimation of the channel gains, that is, the receiver requires the fading amplitude for each diversity branch, and then phase compensation is performed on the signals to add them coherently. On the other hand, EGC has quite acceptable performance and lower implementation complexity than MRC since it only involves a coherent summation of the received signals [23]. To do so, the EGC receiver performs a phase detection and then a compensation stage. Conversely, the MRC receiver requires the same structures as EGC and, additionally, it employs pilot symbols to estimate the fading amplitude. By the above, EGC constitutes an interesting alternative considering the trade-off between performance and implementation complexity, especially for future mobile systems in which tens or hundreds of receiving antennas will be used.

In [24,25], the performance of MRC and EGC systems in generalized fading channels and non-identical Weibull fading channels was evaluated in terms of the outage probability (OP) [26], respectively. More specifically, in [24], the authors derived expressions to evaluate the OP in EGC systems considering Nakagami-*m* fading. Moreover, in [25], the authors indicated that there was no available analytical expression for analyzing the moment-generating-function of the output signal-to-noise ratio (SNR) for EGC receivers operating over Weibull fading channels. As a consequence, they derived approximate expressions for the OP in this scenario. In [27], the authors proposed a multi-tag EGC scheme over Nakagami-*m* fading channels. In particular, this work analyzed a radio frequency identification (RFID) communication scenario. OP expressions were derived using the characteristic function method. Moreover, in [28], the OP for L-branch EGC diversity receivers was evaluated via simulations using the concept of importance sampling (IS) for Rayleigh and Rician fading channels. In [29], the OP and error rate performance of EGC over correlated Beaulieu–Xie fading channels were evaluated, where closed-form asymptotic expressions (lower and upper bounds) were derived for these performance indicators.

Several relaying techniques have been proposed in the literature to improve the performance of wireless communication systems. In [30], the authors developed low-complexity cooperative communication protocols that combat multipath-induced fading. Among them, we highlight the decode-and-forward (DF) protocol, which considers a relay that demodulates the signals and then modulates and re-transmits them to the final receiver (destination) [31]. This process increases the capacity and reliability of user terminals over bad or severe propagation conditions.

The DF protocol has been studied in different scenarios. In [32,33], the performance of wireless systems considering DF relays and Rayleigh fading channels was analyzed in terms of the OP and the average bit error probability (ABEP), respectively. In particular, in [32], an exact closed-form expression for the OP was derived assuming dissimilar fading parameters. Moreover, in [33], the authors derived ABEP expressions for binary differential phase-shift keying, as well as OP expressions for noise-limited systems considering Rayleigh fading channels. In [34], the performance of a DF relay system over Rayleigh fading channels, in which the receivers employ the selection combining technique, was analyzed. For this scenario, an exact closed-form expression to evaluate the OP was derived. In [31], a dual-hop DF relaying system was considered in which the relay and destination nodes were subject to co-channel interference in the presence of Weibull fading. For this scenario, OP expressions based on moment estimators were derived, which were obtained as an infinite summation of Meijer’s G-functions [35] ([Equation (5.3-1)]). In [36], the performance of dual-hop DF cognitive relay networks over independent non-identically distributed (i.n.i.d.) Weibull fading channels was evaluated in terms of the OP, the symbol error probability (SEP), and the ergodic capacity. Integral form expressions were derived for evaluating these key performance indicators (KPIs). In [37], a dual-hop DF system, in which the receivers employ a single antenna, was considered, where one hop was subject to η-μ fading and the other hop was subject to κ-μ fading. Here, SEP, OP, and outage capacity expressions were obtained in terms of infinite series. In [38], the OP of antenna selection schemes in dual-hop DF relay networks over Nakagami-*m* fading channels was investigated, considering that the source, relay, and destination were equipped with multiple antennas. Exact expressions in terms of nested summations were found to evaluate the OP. In [39], a power beacon-assisted cooperative decode-amplify-forward relaying network was analyzed, where the relaying transmission could be selected to remain silent or transmit information employing a DF or amplify-and-forward (AF) protocol based on the channel quality among the source, relay, and destination. For this scenario, closed-form expressions of the OP and the throughput over Rayleigh fading channels were derived. Recently, in [40], the authors considered non-orthogonal multiple access (NOMA) systems that employed dual-hop hybrid optical wireless and radio frequency communications with the aid of a DF relay. In particular, the optical wireless channel underwent exponential-generalized Gamma (EGG) fading, whereas the radio frequency channel underwent Rayleigh fading. Exact closed-form expressions of the OP were obtained in terms of the Mejier-G function, and approximated closed-form expressions of the ergodic capacity were derived in terms of the incomplete Gamma function [41] ([Equation (6.5.3)]). Finally, in [42], an exact closed-form expression for the SNR at the output of NOMA-EGC receivers was derived using a Laplace transform approach for a generalized κ−μ fading channel. Unfortunately, the derived expression was only valid for κ→0, which is equivalent to Nakagami-*m* fading.

Table 1 summarizes the key contributions of the aforementioned works. Based on the literature review, and to the best of the authors’ knowledge, a wireless system that employs dual-hop DF relaying and EGC receivers has not previously been analyzed over Weibull fading channels. This is the primary motivation for this work, where a dual-hop cooperative network operates in which the relay node (R) assists the transmission from a source (S) to the destination (D) using the DF protocol. The relay operates in half-duplex mode, and S does not have a direct link to D due to the presence of deep shadowing. In addition, S and D employ EGC with a different number of receiving antennas. By considering this scenario, the main contributions of this paper are summarized as follows:

- The PDF and the CDF of the SNR at the output of EGC receivers are calculated considering Weibull fading channels.
- Novel exact and asymptotic closed-form expressions to evaluate the OP and the ABEP of dual-hop DF-EGC relaying receivers are also derived, which are easy to manipulate and do not rely on functions that are not built-in in common mathematical software.
- Monte Carlo simulations in different scenarios show the accuracy of the derived expressions. Furthermore, the achievable rate of the considered system is also evaluated via simulations. Useful insights regarding the system performance are obtained from these numerical results.

The remaining sections are organized as follows: Section 2 presents some preliminary results related to the sum of Weibull random variables. Section 3 presents the system and channel models. Exact expressions for calculating the OP and the ABEP of DF-EGC systems are derived in Section 4, where an asymptotic analysis is also carried out. The numerical results are presented in Section 5. Finally, the main conclusions of this work are summarized in Section 6.

In the following, $f_{X}\left(X\right)$ and $F_{X}\left(x\right)$ denote the PDF and the CDF of the random variable *X*, respectively. In addition, E[·] denotes the expectation, P(·) represents the probability operator, $R^{+}$ denotes the set of positive real numbers, and the min(a,b) function returns the argument with the lowest value.

## 2. Preliminaries

The performance analysis of EGC receivers involves the sum of *N* fading envelopes, where *N* is the diversity order, or equivalently, the number of antennas in the array, i.e.,

(1) $$Z=\sum_{n=1}^{N}X_{n},$$

where $X_{n}\in R^{+}$ is a Weibull random variable with PDF given by [43] ([Equation (2)])

(2) $$f_{X_{n}}\left(x_{n}\right)=\frac{k}{x_{n}}{\left(\frac{x_{n}}{\lambda }\right)}^{k}\exp\left[-{\left(\frac{x_{n}}{\lambda }\right)}^{k}\right],\;x_{n}\geq 0,$$

where k>0 and λ>0 represent the shape and scale parameters of the Weibull distribution, respectively.

Assuming that ${\left\{X_{n}\right\}}_{n=1}^{N}$ is a set of i.i.d. Weibull random variables, the PDF of *Z* can be obtained from [19] ([Equation (4)]) as follows:

(3) $$f_{Z}\left(z\right)=z^{-1}k^{N}{\left(\frac{z}{\lambda }\right)}^{Nk}\sum_{i=0}^{\infty }\frac{\delta_{i}z^{ik}}{\Gamma (ik+Nk)},\;z\geq 0,$$

where Γ(·) is the gamma function [41] ([Equation (6.1.1)]) given by

(4) $$\Gamma \left(y\right)=\int_{0}^{\infty }t^{y-1}\exp\left(-t\right)dt,$$

and the coefficients $\delta_{i}$ are obtained recursively employing the following expressions [19] ([Equation (5)])

(5) $$\begin{array}{cc} \delta_{0}= & \Gamma {\left(k\right)}^{N}, \end{array}$$

(6) $$\begin{array}{cc} \delta_{i}= & \frac{1}{i\Gamma (k)}\sum_{\ell =1}^{i}\frac{\delta_{i-\ell }\left(-i+\ell N+\ell \right)\Gamma \left(\ell k+k\right){\left(-{\left(\frac{1}{\lambda }\right)}^{k}\right)}^{\ell }}{\ell !}. \end{array}$$

Although (3) has an infinite summation, according to [19], it is enough to use a suitable finite number of terms to guarantee quite accurate results.

## 3. System and Channel Models

**Figure 1:**
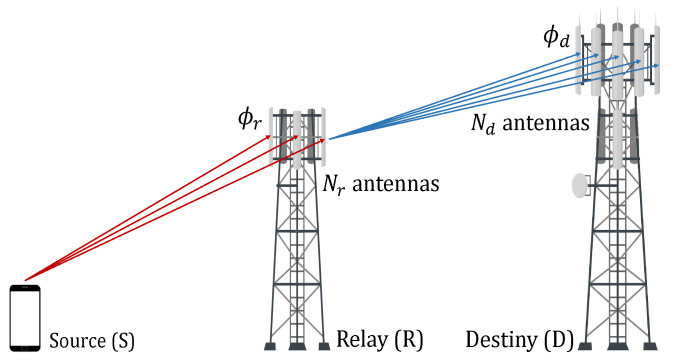
DF-EGC dual-hop relay system model.

Consider a dual-hop cooperative network where R assists the transmission from S to D using the DF protocol, as shown in Figure 1. The relay operates in half-duplex mode, and S does not have a direct link to D due to the presence of deep shadowing. In addition, the nodes R and D employ $N_{r}$ and $N_{d}$ antenna elements, respectively. The impinging received signals at nodes R and D undergo i.i.d. Weibull fading and they are combined using the EGC technique. Moreover, $\phi_{r}$ and $\phi_{d}$ denote the instantaneous SNR at the outputs of the EGC receivers at R and D, respectively.

The diversity branches are affected by additive white Gaussian noise (AWGN). Thus, each noise sample can be modeled by a zero-mean complex Gaussian random variable with variance

(7) $$\sigma_{w}^{2}=\frac{N_{0}}{T_{s}},$$

where $N_{0}$ is the unilateral noise power spectral density and $T_{s}$ is the symbol duration.

## 4. Performance Analysis

In this section, the performance of a DF-EGC relay system is evaluated in terms of the OP and the ABEP for binary modulations.

The methodology used for the analysis is described as follows: The PDF and the CDF of the SNR at the output of EGC systems are first calculated based on the preliminary results given in Section 2. Then, using the CDF expression and considering the operation scheme of DF relay systems, an exact expression to evaluate the OP of DF-EGC systems over Weibull fading is derived. Considering that the region of interest for the operation of wireless systems occurs in high SNR, an asymptotic analysis of the OP expression is performed. In the following, using the PDF- and CDF-derived expressions and using some preliminary results of the BEP for binary modulations over AWGN channels, an exact expression to calculate the ABEP of DF-EGC relay systems is derived. Then, an asymptotic closed-form analysis of this expression is also performed to provide more details about the system behavior. Some insights are obtained from all the derived expressions, which are also validated via Monte Carlo simulations in Section 5.

### 4.1. PDF and CDF of the SNR in EGC Systems

The instantaneous SNR at the output of an EGC receiver can be written as [26] ([Equation (9.51)])

(8) $$\Phi =\frac{1}{N}\frac{P}{\sigma_{w}^{2}}{\left(\;\sum_{n=1}^{N}X_{n}\right)}^{2},$$

where the factor 1/N normalizes the received power per antenna, and *P* is the received power per symbol.

From (1) and (8), the instantaneous SNR for an EGC system can be rewritten as

(9) $$\Phi =\frac{1}{N}\frac{E_{s}}{N_{0}}Z^{2},$$

it was employed (7), where the received energy per symbol is $E_{s}=PT_{s}$, and $E_{s}/N_{0}$ is the normalized SNR per symbol. Since Z≥0, (9) is a monotonic function, with the aid of [44] ([Equation (5.16)]) given by

(10) $$f_{Y}\left(y\right)=\sum_{\ell =1}^{W}\frac{f_{X}\left(x_{\ell }\right)}{|g^{\prime }\left(x_{\ell }\right)|},$$

where *W* is the number of roots of the function y=g(x), *X* is a random variable, g(x) is a function of *X* and $g^{\prime }\left(x\right)$ is the derivative of g(x), and, using (3), it can be shown that the PDF of Φ is given by

(11) $$\begin{array}{c} f_{\Phi }\left(\phi \right)=\;\frac{N}{2}{\left(\frac{E_{s}}{N_{0}}\right)}^{-1}{\left[k{\left(\frac{1}{\lambda }\right)}^{k}\right]}^{N}\sum_{i=0}^{\infty }\frac{\delta_{i}}{\Gamma \left(ik+Nk\right)}{\left(\frac{N\phi }{E_{s}/N_{0}}\right)}^{\frac{k}{2}\left(i+N\right)-1},\;\phi \geq 0. \end{array}$$

**Figure 2:**
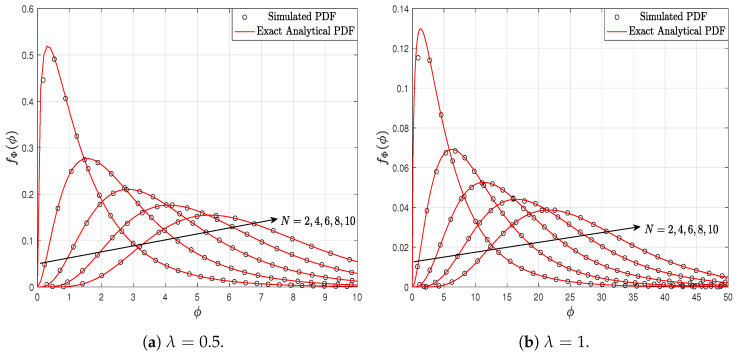
PDF of the SNR for an EGC system, parameterized by *N*, and λ considering k=1.5.

Figure 2 shows the PDF of the SNR for an EGC system, parameterized by different values of *N* and considering k=1.5 in the Weibull fading. Figure 2a assumes λ=0.5 and Figure 2b assumes λ=1. This change in the λ parameter causes the PDF to have different behaviors. Thus, in the case of λ=0.5, it can be observed that there is a greater probability that the random variable ϕ assumes small values. This is evidenced in the horizontal axis of the figures, where the horizontal axis of Figure 2a has values up to 10 and the horizontal axis of Figure 2b has values up to 50. In the figures, it should be noted also that the simulated PDF results agree with the theoretical PDF results in all the scenarios. In particular, the analytical PDFs are generated using 80 terms in the summation of (11).

From (11), the CDF of Φ can be obtained as

(12) $$\begin{array}{cc} F_{\Phi }\left(\phi \right) & =\int_{0}^{\phi }f_{\Phi }\left(x\right)dx\\ \; & =\sum_{i=0}^{\infty }\frac{\delta_{i}}{\Gamma \left(ik+Nk\right)}\frac{k^{N-1}\lambda^{-kN}}{\left(i+N\right)}{\left(\frac{N\phi }{E_{s}/N_{0}}\right)}^{\frac{k}{2}\left(i+N\right)}. \end{array}$$

### 4.2. Outage Probability

The instantaneous SNR for dual-hop DF relay systems can be written as [31]

(13) $$\begin{array}{c} \phi_{\mathrm{df}}=\min\left(\phi_{r},\phi_{d}\right), \end{array}$$

where $\phi_{r}$ and $\phi_{d}$ were defined in Section 3.

Since $\phi_{r}$ and $\phi_{d}$ are i.i.d. random variables, the OP for dual-hop DF relay systems can be calculated as

(14) $$\begin{array}{cc} \mathrm{OP} & =P\left[\min\left(\phi_{r},\phi_{d}\right)<\phi_{\mathrm{th}}\right]\\ & =1-P\left[\phi_{r}>\phi_{\mathrm{th}},\phi_{d}>\phi_{\mathrm{th}}\right]\\ & =1-\left(1-P\left[\phi_{r}\leq \phi_{\mathrm{th}}\right]\right)\left(1-P\left[\phi_{d}\leq \phi_{\mathrm{th}}\right]\right)\\ \; & =1-\left[1-F_{\Phi_{r}}\left(\phi_{\mathrm{th}}\right)\right]\left[1-F_{\Phi_{d}}\left(\phi_{\mathrm{th}}\right)\right], \end{array}$$

where we have used [44] ([Equation (6.81)]), given by

(15) $$\begin{array}{cc} F_{W}\left(w\right) & =1-P(x>w,y>w)\\ \; & =F_{X}\left(w\right)+F_{Y}\left(w\right)-F_{X}\left(w\right)F_{Y}\left(w\right), \end{array}$$

where w,x, and *y* are random variables. In particular, *x* and *y* are independent random variables. Moreover, in (14), $\phi_{\mathrm{th}}$ is a threshold value for the SNR in order to guarantee an adequate system performance, and $F_{\Phi_{r}}\left(\phi_{r}\right)$ and $F_{\Phi_{d}}\left(\phi_{d}\right)$ are the CDF of the received SNR at R and D, respectively.

By considering that R and D can employ a different number of receiving antennas, and as our system model considers EGC receivers at both R and D, then $F_{\Phi_{r}}\left(\phi_{r}\right)$ and $F_{\Phi_{d}}\left(\phi_{d}\right)$ are given by (12), and *N* is replaced by $N_{r}$ and $N_{d}$, as appropriate. In addition, the subscripts *r* and *d* are used to differentiate the fading channel parameters related to the links S → R and R → D, respectively. Accordingly, the OP for the considered DF-EGC system can be written as

(16) $$\begin{array}{c} \mathrm{OP}=1-\left[1-\sum_{i=0}^{\infty }\delta_{i}\Upsilon_{r,i}{\left(\frac{\phi_{\mathrm{th}}}{E_{s}/N_{0}}\right)}^{\frac{1}{2}k_{r}\left(i+N_{r}\right)}\right]\left[1-\sum_{i=0}^{\infty }\delta_{i}\Upsilon_{d,i}{\left(\frac{\phi_{\mathrm{th}}}{E_{s}/N_{0}}\right)}^{\frac{1}{2}k_{d}\left(i+N_{d}\right)}\right], \end{array}$$

where

(17) $$\Upsilon_{x,i}=\frac{1}{\Gamma \left(ik_{x}+N_{x}k_{x}\right)}\frac{k_{x}^{N_{x}-1}\lambda_{x}^{-k_{x}N_{x}}}{\left(i+N_{x}\right)}N_{x}^{\frac{1}{2}k_{x}\left(i+N_{x}\right)},$$

for x∈{r,d}.

Now, an asymptote for the OP expression in the high SNR region is derived. It can be observed that, as $E_{s}/N_{0}\to \infty$, the most dominant term in the infinite series of (16) appears for i=0. Thus, by considering only this term, an asymptotic expression for the OP can be obtained as

(18) $$\begin{array}{cc} \mathrm{OP} & ≃\;1-\left[1-\delta_{0}\Upsilon_{r,0}{\left(\frac{\phi_{\mathrm{th}}}{E_{s}/N_{0}}\right)}^{\frac{1}{2}k_{r}N_{r}}\;\right]\left[1-\delta_{0}\Upsilon_{d,0}{\left(\frac{\phi_{\mathrm{th}}}{E_{s}/N_{0}}\right)}^{\frac{1}{2}k_{d}N_{d}}\right]\\ \; & ≃\;\delta_{0}\Upsilon_{r,0}{\left(\frac{\phi_{\mathrm{th}}}{E_{s}/N_{0}}\right)}^{\frac{1}{2}k_{r}N_{r}}+\delta_{0}\Upsilon_{d,0}{\left(\frac{\phi_{\mathrm{th}}}{E_{s}/N_{0}}\right)}^{\frac{1}{2}k_{d}N_{d}}-\delta_{0}^{2}\Upsilon_{r,0}\Upsilon_{d,0}{\left(\frac{\phi_{\mathrm{th}}}{E_{s}/N_{0}}\right)}^{\frac{1}{2}\left(k_{r}N_{r}+k_{d}N_{d}\right)}, \end{array}$$

where the last term in the sum is negligible as $E_{s}/N_{0}\to \infty$ because the exponent of the $E_{s}/N_{0}$ is greater than that of the first two terms.

Moreover, the dominant term in (18) is the one in which the product $k_{x}N_{x}$ is smallest, for x∈{r,d}. Therefore, the asymptote for the OP in the high $E_{s}/N_{0}$ region can be written as

(19) $$\mathrm{OP}≃\;\Omega {\left(\frac{\phi_{\mathrm{th}}}{E_{s}/N_{0}}\right)}^{\xi },$$

where

(20) $$\Omega =\left\{\begin{array}{cc} \delta_{0}\Upsilon_{r,0}, & k_{r}N_{r}\leq k_{d}N_{d}\\ \delta_{0}\Upsilon_{d,0}, & \mathrm{otherwise} \end{array}\right.$$

is named as the coding gain, and

(21) $$\xi =\min\left\{\frac{1}{2}k_{r}N_{r},\frac{1}{2}k_{d}N_{d}\right\}$$

is the system diversity order.

### 4.3. Average Bit Error Probability

In (13), $\phi_{r}$ and $\phi_{d}$ are i.i.d. random variables; thus, from (15), it can be shown that the PDF of $\phi_{\mathrm{df}}$, given by (13), is equal to

(22) $$f_{\Phi_{\mathrm{df}}}\left(\phi_{\mathrm{df}}\right)=f_{\Phi_{r}}\left(\phi_{\mathrm{df}}\right)\left[1-F_{\Phi_{d}}\left(\phi_{\mathrm{df}}\right)\right]+f_{\Phi_{d}}\left(\phi_{\mathrm{df}}\right)\left[1-F_{\Phi_{r}}\left(\phi_{\mathrm{df}}\right)\right].$$

Similarly to the OP analysis, $F_{\Phi_{r}}\left(\phi_{r}\right)$ and $F_{\Phi_{d}}\left(\phi_{d}\right)$ are given by (12), *N* is replaced by $N_{r}$ and $N_{d}$, as appropriate, and the subscripts *r* and *d* are used to differentiate the fading channel parameters related to the links S → R and R → D, respectively. In addition, $f_{\Phi_{r}}\left(\phi_{r}\right)$ and $f_{\Phi_{d}}\left(\phi_{d}\right)$ are given by (11) and the same aspects as in the case of the CDF are considered. Therefore, (22) can be rewritten as

(23) $$\begin{array}{cc} f_{\Phi_{\mathrm{df}}}\left(\phi_{\mathrm{df}}\right)= & \;\frac{1}{2}\alpha_{r}\sum_{i=0}^{\infty }\zeta_{r,i}\phi_{\mathrm{df}}^{\beta_{r,i}-1}\left[1-\frac{\alpha_{d}}{k_{d}}\sum_{j=0}^{\infty }\frac{\zeta_{d,j}}{j+N_{d}}\phi_{\mathrm{df}}^{\beta_{d,j}}\right]\\ \; & +\frac{1}{2}\alpha_{d}\sum_{i=0}^{\infty }\zeta_{d,i}\phi_{\mathrm{df}}^{\beta_{d,i}-1}\left[1-\frac{\alpha_{r}}{k_{r}}\sum_{j=0}^{\infty }\frac{\zeta_{r,j}}{j+N_{r}}\phi_{\mathrm{df}}^{\beta_{r,j}}\right], \end{array}$$

where

(24) $$\alpha_{x}={\left[k_{x}{\left(\frac{1}{\lambda_{x}}\right)}^{k_{x}}\right]}^{N_{x}},$$

(25) $$\beta_{x,i}=\frac{1}{2}k_{x}\left(i+N_{x}\right),$$

and

(26) $$\zeta_{x,i}=\frac{\delta_{i}}{\Gamma (ik_{x}+N_{x}k_{x})}{\left(\frac{N_{x}}{E_{s}/N_{0}}\right)}^{\beta_{x,i}},$$

for x∈{r,d}.

Then, the ABEP can be calculated as [1]

(27) $$\bar{P_{b}}=\int_{0}^{\infty }P_{b}\left(\phi_{\mathrm{df}}\right)f_{\Phi_{\mathrm{df}}}\left(\phi_{\mathrm{df}}\right)d\phi_{\mathrm{df}},$$

where $P_{b}\left(\phi_{\mathrm{df}}\right)$ is the BEP in AWGN channels conditioned on the instantaneous value of $\phi_{\mathrm{df}}$. For binary modulations, it is known that [26] ([Equation (9.2)])

(28) $$P_{b}\left(\phi_{\mathrm{df}}\right)=\frac{1}{2}\mathrm{erfc}\left(\sqrt{a\phi_{\mathrm{df}}}\right),$$

where *a* depends on the modulation scheme: a=1 is used for coherent binary-phase-shift-keying (BPSK), a=1/2 is employed for coherent orthogonal binary-frequency-shift-keying (BFSK), and a=0.715 for coherent BFSK with minimum correlation.

From (23)–(28), the ABEP is obtained as

(29) $$\begin{array}{cc} \bar{P_{b}}= & \;\frac{1}{4}\alpha_{r}\sum_{i=0}^{\infty }\zeta_{r,i}\left[\int_{0}^{\infty }\mathrm{erfc}\left(\sqrt{a\phi_{\mathrm{df}}}\right)\phi_{\mathrm{df}}^{\beta_{r,i}-1}d\phi_{\mathrm{df}}\right.\\ \; & \;\left.-\frac{\alpha_{d}}{k_{d}}\sum_{j=0}^{\infty }\frac{\zeta_{d,j}}{j+N_{d}}\int_{0}^{\infty }\mathrm{erfc}\left(\sqrt{a\phi_{\mathrm{df}}}\right)\phi_{\mathrm{df}}^{\beta_{r,i}+\beta_{d,j}-1}d\phi_{\mathrm{df}}\right]\\ \; & +\frac{1}{4}\alpha_{d}\sum_{i=0}^{\infty }\zeta_{d,i}\left[\int_{0}^{\infty }\mathrm{erfc}\left(\sqrt{a\phi_{\mathrm{df}}}\right)\phi_{\mathrm{df}}^{\beta_{d,i}-1}d\phi_{\mathrm{df}}\right.\\ \; & \;\left.-\frac{\alpha_{r}}{k_{r}}\sum_{j=0}^{\infty }\frac{\zeta_{r,j}}{j+N_{r}}\int_{0}^{\infty }\mathrm{erfc}\left(\sqrt{a\phi_{\mathrm{df}}}\right)\phi_{\mathrm{df}}^{\beta_{d,i}+\beta_{r,j}-1}d\phi_{\mathrm{df}}\right]. \end{array}$$

With the aid of [45] ([Equation (4.1.18)]), a change of variables, and after some algebraic manipulations, (29) can be rewritten as

(30) $$\begin{array}{cc} \bar{P_{b}}= & \frac{1}{4\sqrt{\pi }}\left\{\alpha_{r}\sum_{i=0}^{\infty }\zeta_{r,i}\left[\frac{\Gamma \left(\beta_{r,i}+1/2\right)}{a^{\beta_{r,i}}\beta_{r,i}}-\frac{\alpha_{d}}{k_{d}}\sum_{j=0}^{\infty }\frac{\zeta_{d,j}}{\left(j+N_{d}\right)}\frac{\Gamma (\beta_{r,i}+\beta_{d,j}+1/2)}{a^{\beta_{r,i}+\beta_{d,j}}\left(\beta_{r,i}+\beta_{d,j}\right)}\right]\right.\\ \; & \;\left.+\alpha_{d}\sum_{i=0}^{\infty }\zeta_{d,i}\left[\frac{\Gamma \left(\beta_{d,i}+1/2\right)}{a^{\beta_{d,i}}\beta_{d,i}}-\frac{\alpha_{r}}{k_{r}}\sum_{j=0}^{\infty }\frac{\zeta_{r,j}}{\left(j+N_{r}\right)}\frac{\Gamma (\beta_{r,j}+\beta_{d,i}+1/2)}{a^{\beta_{r,j}+\beta_{d,i}}\left(\beta_{r,j}+\beta_{d,i}\right)}\right]\right\}. \end{array}$$

In (30), as $E_{s}/N_{0}\to \infty$, the most dominant terms in the infinite series appear for i=0 and j=0. Thus, an asymptotic expression for the ABEP can be calculated considering only these terms; then, we obtain

(31) $$\begin{array}{cc} \bar{P_{b}}≃ & \frac{1}{4\sqrt{\pi }}\left\{\alpha_{r}\zeta_{r,0}\left[\frac{\Gamma \left(\beta_{r,0}+1/2\right)}{a^{\beta_{r,0}}\beta_{r,0}}-\frac{\alpha_{d}}{k_{d}}\frac{\zeta_{d,0}}{N_{d}}\frac{\Gamma (\beta_{r,0}+\beta_{d,0}+1/2)}{a^{\beta_{r,0}+\beta_{d,0}}\left(\beta_{r,0}+\beta_{d,0}\right)}\right]\right.\\ \; & \;\left.+\alpha_{d}\zeta_{d,0}\left[\frac{\Gamma \left(\beta_{d,0}+1/2\right)}{a^{\beta_{d,0}}\beta_{d,0}}-\frac{\alpha_{r}}{k_{r}}\frac{\zeta_{r,0}}{N_{r}}\frac{\Gamma (\beta_{r,0}+\beta_{d,0}+1/2)}{a^{\beta_{r,0}+\beta_{d,0}}\left(\beta_{r,0}+\beta_{d,0}\right)}\right]\right\}. \end{array}$$

From (26), the terms in (31) with the product $\zeta_{r,0}\zeta_{d,0}$ have the factor ${\left(E_{s}/N_{0}\right)}^{-\frac{1}{2}\left(k_{r}N_{r}+k_{d}N_{d}\right)}$, which are negligible with respect to the terms with the factors ${\left(E_{s}/N_{0}\right)}^{-\frac{1}{2}k_{r}N_{r}}$ or ${\left(E_{s}/N_{0}\right)}^{-\frac{1}{2}k_{d}N_{d}}$ as $E_{s}/N_{0}$ tends to infinity. Under this premise, we can take the most significative terms in (31) and obtaining

(32) $$\begin{array}{c} \bar{P_{b}}≃\frac{1}{4\sqrt{\pi }}\left\{\alpha_{r}\zeta_{r,0}\frac{\Gamma \left(\beta_{r,0}+1/2\right)}{a^{\beta_{r,0}}\beta_{r,0}}+\alpha_{d}\zeta_{d,0}\frac{\Gamma \left(\beta_{d,0}+1/2\right)}{a^{\beta_{d,0}}\beta_{d,0}}\right\}. \end{array}$$

Considering again (26), and as $E_{s}/N_{0}\to \infty$, the most dominant term in (32) is the one in which the product $k_{x}N_{x}$ is smallest for x∈{r,d}. Hence, after some algebraic manipulations, and using (24)–(26), the asymptote for the ABEP in the high $E_{s}/N_{0}$ region can be written as

(33) $$\bar{P_{b}}≃\;\Psi {\left(\frac{1}{aE_{s}/N_{0}}\right)}^{\xi },$$

where

(34) $$\Psi =\left\{\begin{array}{cc} \frac{\delta_{0}}{2\sqrt{\pi }}\Gamma \left(\frac{1}{2}\left(K_{r}N_{r}+1\right)\right)\Upsilon_{r,0}, & k_{r}N_{r}\leq k_{d}N_{d}\\ \frac{\delta_{0}}{2\sqrt{\pi }}\Gamma \left(\frac{1}{2}\left(K_{d}N_{d}+1\right)\right)\Upsilon_{d,0}, & \mathrm{otherwise} \end{array}\right.$$

is the coding gain in the ABEP asymptote, where $\Upsilon_{x,i}$ is given by (17), and ξ is the diversity gain, which is given by (21). Thus, the DF-EGC relay system diversity is not only determined by the receiver with the least number of antennas (R or D), but also by the Weibull distribution shape parameter *k* of each link, which plays an important role in the system performance.

### 4.4. Achievable Rate

The achievable rate in bps/Hz (bits per second/Hertz) of DF-EGC systems can be calculated considering Shannon’s capacity expression for signal transmission over AWGN channels [46]; that is,

(35) $$\begin{array}{cc} C(\phi_{\mathrm{df}}) & =\log_{2}\left(1+\phi_{\mathrm{df}}\right)\\ \; & =\log_{2}\left(1+\min(\phi_{r},\phi_{d})\right), \end{array}$$

where we have employed (13). The mean value of the achievable rate, i.e., $E[C\left(\phi_{\mathrm{df}}\right)]$, as well as the OP and the ABEP, are analyzed employing numerical results in the following section.

## 5. Numerical Results and Discussions

In this section, the performance of DF-EGC relay systems is analyzed in terms of the OP, the ABEP, and the mean achievable rate in different scenarios. For the OP and the ABEP, the exact analytical results are obtained employing 80 terms in the summations of (16) and (30), as appropriate. Monte Carlo simulations with $10^{7}$ trials validate the analytical results.

### 5.1. Outage Probability Results

**Figure 3:**
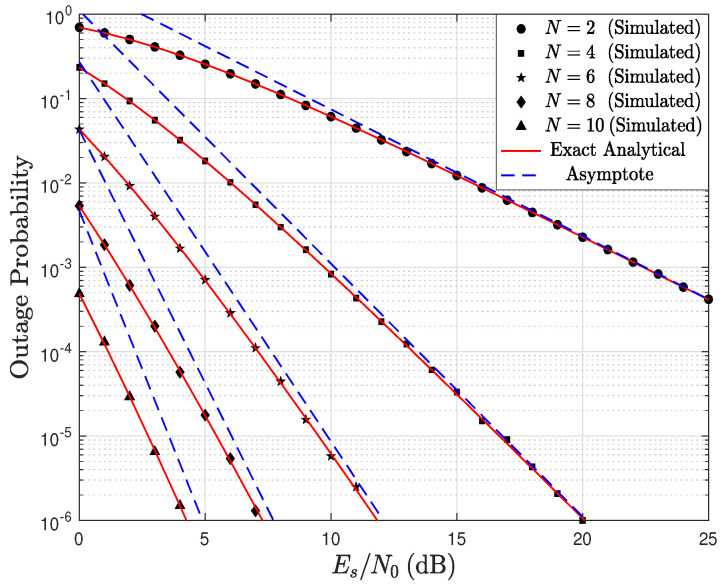
OP as a function of $E_{s}/N_{0}$, parameterized by $N=N_{r}=N_{d}$, and considering $k_{r}=k_{d}=1.5$, and $\lambda_{r}=\lambda_{d}=1$.

Figure 3 shows the OP as a function of the $E_{s}/N_{0}$ ratio parameterized by the number of receiving antennas. In this first scenario, it is assumed that the number of antennas at R and at D are equal, i.e., $N_{r}=N_{d}=N$. Additionally, it is assumed that the channel conditions for the links S→R and R→D are also equal; therefore, $k_{r}=k_{d}=1.5$ and, $\lambda_{r}=\lambda_{d}=1$. In the figure, as $E_{s}/N_{0}$ increases, the OP decreases, which is an expected result. In addition, when the number of antennas increases, the OP decreases since the additional number of antennas guarantees more diversity in the system. Moreover, it can be observed that the analytical results agree perfectly with the simulation results in all the scenarios. In particular, the exact analytical curve is obtained employing (16) and the asymptote is calculated via (19).

**Figure 4:**
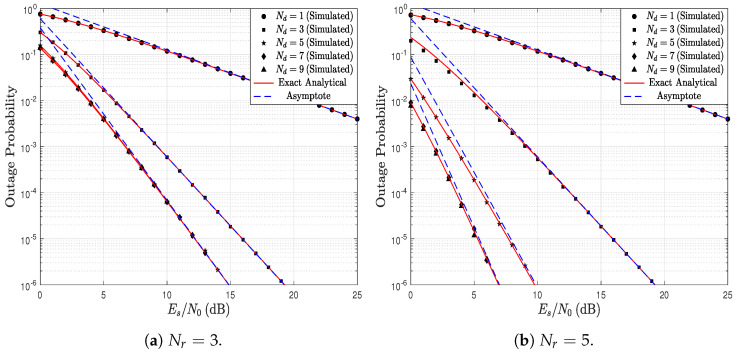
OP as a function of $E_{s}/N_{0}$, parameterized by $N_{r}$ and $N_{d}$, considering $k_{r}=2.5$, $k_{d}=2$, and $\lambda_{r}=\lambda_{d}=1$.

Figure 4 shows the OP as a function of the $E_{s}/N_{0}$ ratio parameterized by $N_{r}$ and $N_{d}$. It is assumed that the link S → R is over different propagation conditions than the link R → D; thus, $k_{r}=2.5$ and $k_{d}=2$. In addition, it is considered that $\lambda_{r}=\lambda_{d}=1$. In Figure 4a, where $N_{r}=3$, it can be observed that, when the number of antennas in D increases, the system performance improves. However, it can be observed that when D has more than 5 antennas, the OP does not decrease. From (19) and (21), the system performance is dictated by the link in which the product between the Weibull distribution parameter *k* and the number of antennas is smallest. Consequently, the link S → R dictates the performance in this scenario, and increasing the number of antennas in D does not reduce the OP. In counterpart, in Figure 3, it was noticed that increasing the number of antennas reduces the OP; however, in that scenario the number of antennas increased simultaneously in both R and D. Figure 4b is similar to Figure 4a, but $N_{r}=5$ is employed. When comparing both figures, it can be observed that lower OP values are obtained in Figure 4b. This occurs because the number of antennas in R increases from $N_{r}=3$ to $N_{r}=5$. Nevertheless, similarly to the previous scenario, the performance is dictated by the link S → R. Therefore, it can be observed that, from a number of antennas greater than $N_{d}=7$, the system performance does not improve.

**Figure 5:**
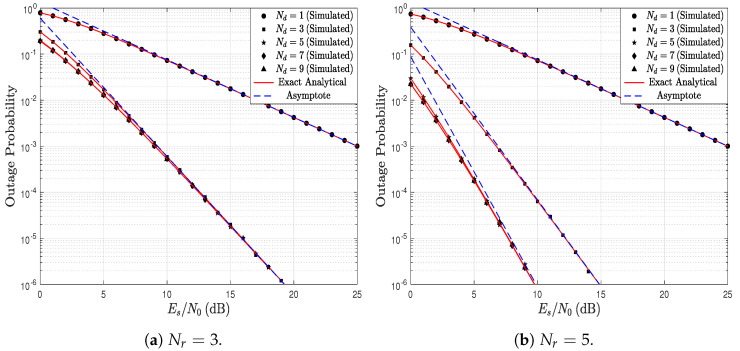
OP as a function of $E_{s}/N_{0}$, parameterized by $N_{r}$ and $N_{d}$, considering $k_{r}=2$, $k_{d}=2.5$, and $\lambda_{r}=\lambda_{d}=1$.

Figure 5 shows the OP as a function of the $E_{s}/N_{0}$ ratio parameterized by $N_{d}$ and $N_{r}$. In this case, it is considered that $k_{r}=2$ and $k_{d}=2.5$. In Figure 5a, it can be observed that, when $N_{d}=5$ antennas, the performance improves slightly in the low SNR region when compared to the scenario $N_{d}=3$ antennas, but the OP does not improve in the high SNR region when compared to the same scenario. A similar behavior is observed in Figure 5b when $N_{d}=7$. This OP behavior was not observed in Figure 4 where $k_{r}>k_{d}$. Hence, these results evidence that the fading channel parameters (*k* and λ) have a significant impact on the DF-EGC system performance. Thus, increasing the number of receiving antennas at R or D does not necessarily guarantee that the OP is reduced based on the propagation conditions.

**Figure 6:**
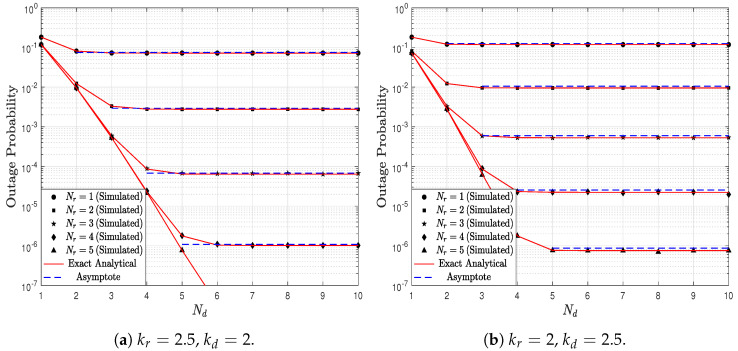
OP as a function of $N_{d}$, parameterized by $N_{r}$, $k_{r}$ and $k_{d}$, considering $E_{s}/N_{0}=10$ dB, and $\lambda_{r}=\lambda_{d}=1$.

Figure 6 shows the OP as a function of $N_{d}$, parameterized by $N_{r}$, $k_{r}$ and $k_{d}$, considering $E_{s}/N_{0}=10$ dB and $\lambda_{r}=\lambda_{d}=1$. It is interesting to observe that increasing the number of antennas in D only ensures a higher performance improvement when the number of antennas in R is also increased. Thus, it can be observed that, if the number of antennas in R is maintained fixed, and the number of antennas in D is increased, then the OP decreases up to a certain value, and then it no longer decreases even though $N_{d}$ increases. Therefore, the number of antennas in both EGC receivers must be properly selected in order to maintain an adequate trade-off between cost and performance. Finally, by comparing Figure 6a,b, it can be observed that, for the same number of antennas at R and D, the scenario of Figure 6a generates lower values of OP. For a better understanding of this result, consider the case where $N_{r}=3$ and $N_{d}=4$. In the scenario of Figure 6a, we have that $N_{r}k_{r}=7.5$ and that $N_{d}k_{d}=8$; as a consequence, from (21), the system diversity order is ξ=3.75. On the other hand, in the scenario of Figure 6b, we have that $N_{r}k_{r}=6$ and $N_{d}k_{d}=10$, then ξ=3. Hence, as the scenario of Figure 6a ensures a higher diversity order, it also ensures a lower OP.

### 5.2. Average Bit Error Probability Results

**Figure 7:**
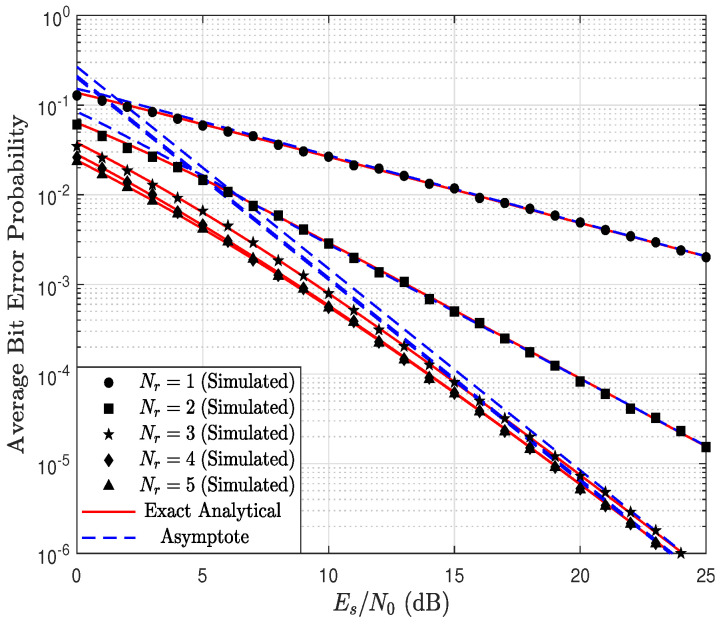
ABEP as a function of $E_{s}/N_{0}$, parameterized by $N_{r}$ considering $N_{d}=5$, $k_{r}=1.5$, $k_{d}=0.9$, $\lambda_{r}=1.4$, $\lambda_{d}=1$ and a=1.

Figure 7 shows the ABEP as a function of the $E_{s}/N_{0}$ ratio, parameterized by $N_{r}$ considering $N_{d}=5$, $k_{r}=1.5$, $k_{d}=0.9$, $\lambda_{r}=1.4$, $\lambda_{d}=1$ and BPSK modulation, i.e, a=1. Notice the accuracy of the exact and asymptotic theoretical results, obtained via (30) and (33), respectively, with the simulated results. In addition, it is observed that, as $N_{r}$ increases, the ABEP decreases. However, as the value of $N_{r}$ approaches the value of $N_{d}$, the improvement in performance becomes smaller. In particular, when $N_{r}=N_{d}=5$, the system performance is dictated by the link R → D, since the product $k_{d}N_{d}$ is less than the product $k_{r}N_{r}$. Therefore, despite the value of $N_{r}$ being increased, the system diversity, or equivalently the system performance, is dictated by the product $k_{d}N_{d}$. Thus, increasing $N_{r}$ does not improve the ABEP. This can be verified analytically through (21) and (33), where it is determined that the system diversity order is equal to 2.25 when $N_{d}=5$. This produces a saturation in the ABEP despite the fact that $N_{r}$ increases.

**Figure 8:**
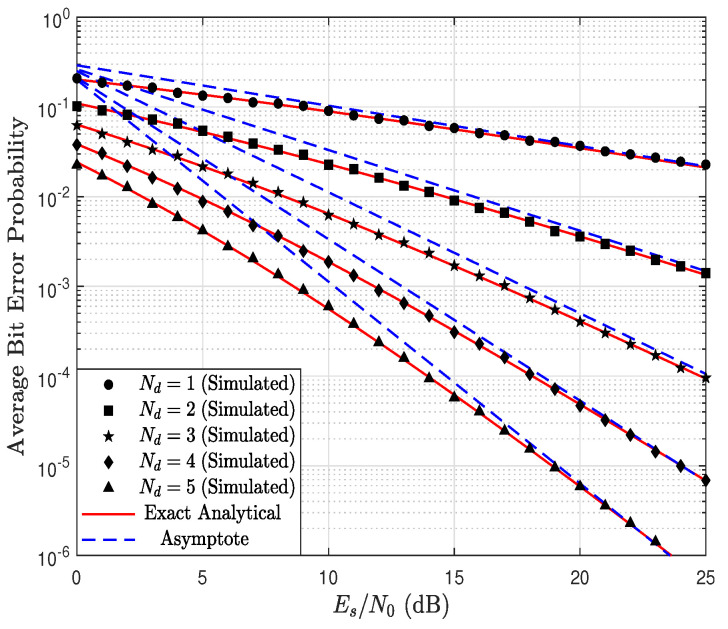
ABEP as a function of $E_{s}/N_{0}$, parameterized by $N_{d}$ considering $N_{r}=5$, $k_{r}=1.5$, $k_{d}=0.9$, $\lambda_{r}=1.4$, $\lambda_{d}=1$, and a=1.

Figure 8 shows the ABEP as a function of $E_{s}/N_{0}$, parameterized by $N_{d}$ considering $N_{r}=5$, $k_{r}=1.5$, $k_{d}=0.9$, $\lambda_{r}=1.4$, $\lambda_{d}=1$ and a=1. In this case, the system performance for $N_{r}\leq 5$ is dictated by the link R → D since $k_{d}N_{d}<k_{r}N_{r}$. As a consequence, increasing $N_{d}$ up to 5 improves the system’s performance; this system behavior will be maintained as long as $k_{d}N_{d}<k_{r}N_{r}$. Therefore, the results of Figure 7 and Figure 8 indicate that increasing the number of antennas excessively in the relay or in the BS does not necessarily improve the system performance.

**Figure 9:**
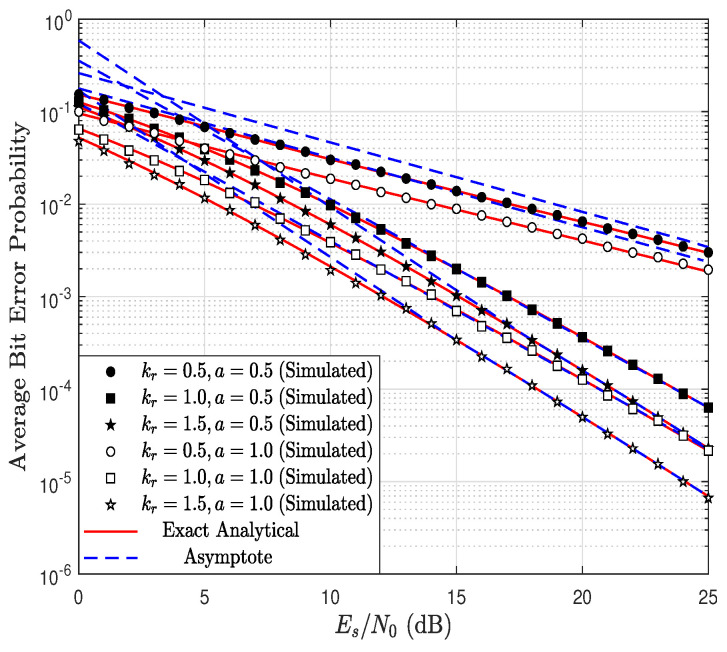
ABEP as a function of $E_{s}/N_{0}$, parameterized by $k_{r}$ and *a* considering $N_{r}=3$, $N_{d}=4$, $k_{d}=0.9$, $\lambda_{r}=1.4$, and $\lambda_{d}=1$.

Figure 9 shows the ABEP as a function of $E_{s}/N_{0}$, parameterized by $k_{r}$ and *a* considering $N_{r}=3$, $N_{d}=4$, $k_{d}=0.9$, $\lambda_{r}=1.4$ and $\lambda_{d}=1$. Thus, this figure indicates the impact that the fading channel affecting the link S → R has on the system performance. In addition, a=1 is used for BPSK and a=1/2 is used for BFSK. It can be observed that, for both modulations, the theoretical results are in full agreement with the simulated results, and, as expected, BPSK has better performance than BFSK for the same value of $k_{r}$. It can be observed that, as $k_{r}$ increases, the ABEP is reduced since the system diversity order increases. Interestingly, there is a greater improvement when $k_{r}$ increases from 0.5 to 1 than when it increases from 1 to 1.5. In the first case, the performance is dictated by the link S → R, given that $k_{r}N_{r}<k_{d}N_{d}$. On the other hand, when $k_{r}$ increases to 1.5, then the performance is now governed by the link R → D because $k_{r}N_{r}>k_{d}N_{d}$.

### 5.3. Mean Achievable Rate Results

Finally, in this section, the performance of DF-EGC systems is evaluated in terms of the mean achievable rate.

**Figure 10:**
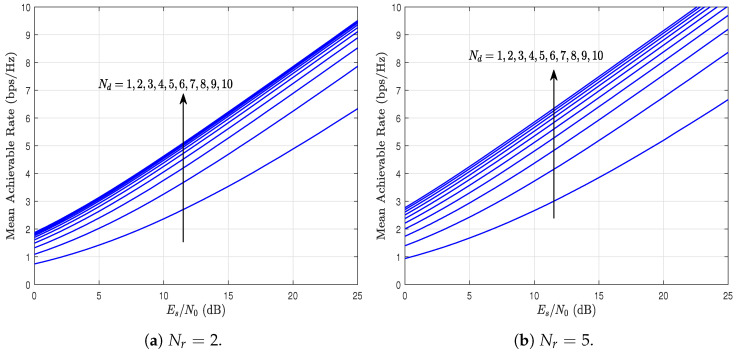
Mean achievable rate as a function of $E_{s}/N_{0}$, parameterized by $N_{r}$, and $N_{d}$ considering $k_{r}=1.5$, $k_{d}=0.9$, $\lambda_{r}=1.4$, and $\lambda_{d}=1$.

Figure 10 shows the mean achievable rate as a function of the $E_{s}/N_{0}$ ratio, parameterized by $N_{r}$ and $N_{d}$ considering $k_{r}=1.5$, $k_{d}=0.9$, $\lambda_{r}=1.4$, and $\lambda_{d}=1$. It can be observed that, as the $E_{s}/N_{0}$ ratio increases, the achievable rate also increases, which is an expected result. Moreover, as $N_{d}$ increases, the mean achievable rate also increases. This occurs because the diversity at destiny increases. However, by comparing Figure 10a,b, it can be observed that the achievable rate increases more slowly when $N_{r}=2$. In fact, in this scenario, a saturation point of the achievable rate is observed; that is, from a certain value of $N_{d}$, the achievable rate no longer increases (or increases very slowly) despite the increase in $N_{d}$. Thus, similar to what was observed in the previous analysis, the performance of the DF-EGC system is given by the link in which the product $k_{x}N_{x}$ is greater for x∈{r,d}. As a result, the saturation point of the rate when $N_{d}$ is increased is higher in the case of Figure 10b, when $N_{r}=5$.

**Figure 11:**
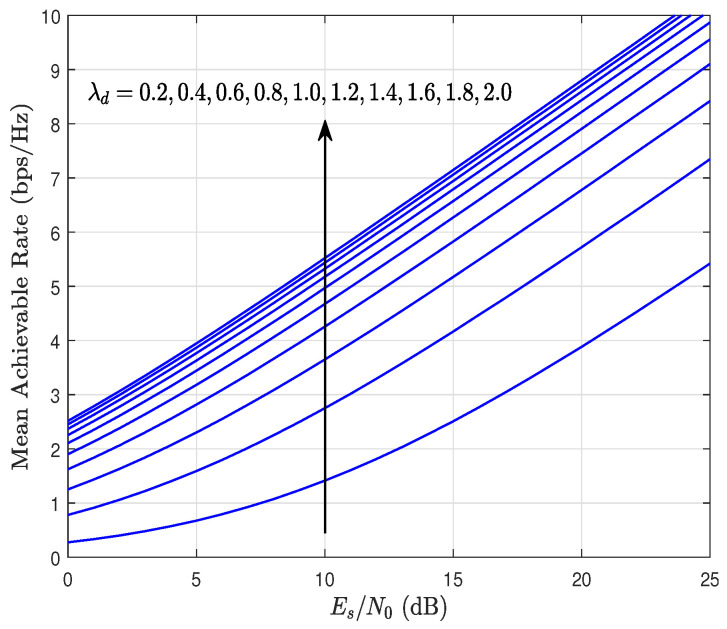
Mean achievable rate as a function of $E_{s}/N_{0}$, parameterized by $\lambda_{d}$ considering $N_{r}=N_{d}=4$, $k_{r}=1.5$, $k_{d}=0.9$, and $\lambda_{r}=1.4$.

Figure 11 shows the mean achievable rate as a function of $E_{s}/N_{0}$, parameterized by $\lambda_{d}$ considering $N_{r}=N_{d}=4$, $k_{r}=1.5$, $k_{d}=0.9$, and $\lambda_{r}=1.4$. Thus, this last figure shows the impact that the λ fading parameter has on the system performance. In particular, the parameter associated with the link R → D is considered. Although this parameter of the Weibull distribution is not directly related to the diversity of the system, it is observed that increasing $\lambda_{d}$ also increases the mean achievable rate. To explain this behavior, Equations (19) and (33) can be considered, where the factor $\Upsilon_{x,i}$ appears, which depends on $\lambda_{x}$, for x∈{r,d}. Therefore, an increase in $\lambda_{d}$ allows for improving the coding gain, which translates into better system performance. More specifically, this implies a lower OP, a lower ABEP, and, in the case of Figure 11, a higher achievable rate.

## 6. Conclusions

The performance of a DF-EGC dual-hop relay system was analyzed over the presence of Weibull fading channels, which is a scenario that has not been previously considered in the literature. In addition, unlike other previous works that considered Weibull fading in diverse scenarios and in which approximate or integral expressions were obtained to calculate some performance indicators, this work has presented exact and asymptotic closed-form expressions to evaluate the OP and the ABEP, which are a function of the fading parameters and the number of antennas at the relay and at the destiny. In addition, the derived expressions can be easily evaluated using widely available computing software, such as Matlab or Mathematica.

The analytical modeling showed that the system diversity is given by the link (S → R or R → D), where the product between the Weibull fading parameter *k* and the number of receiving antennas is smaller, which is a novel result. Thus, increasing the number of receiving antennas at R or D does not necessarily guarantee that the OP or the ABEP are reduced. For this reason, the expressions derived in this work are a helpful tool for designing DF-ECG systems and ensuring an adequate trade-off between cost and performance.

Finally, it is important to note that the ABEP for other modulation schemes, such as quadrature amplitude modulation (QAM), can be easily obtained based on the results presented in this work. In addition, an option for future research is the performance analysis of other techniques, such as by using the amplify-and-forward protocol over Weibull fading, or by considering other combination schemes, such as MRC.

## Figures and Tables

**Table 1:** Related work summary.

Key Contributions	Previous Works								[P]
	[2,3,4]	[5,6]	[21,22]	[27,28,29]	[30,31]	[32,33]	[38,39]	[40]	
Performance evaluation in Rayleigh fading	X				X	X		X	X
Performance evaluation in generalized fading	X	X		X	X		X		X
Exact and asymptotic closed-form expressions for different KPIs	X	X	X	X		X	X		X
Diversity techniques			X	X				X	X
Cooperative communications					X	X	X	X	X

[P] = Proposal presented in this work.

## Data Availability

Some algorithms developed in this work can be found in this https://1drv.ms/f/s!AsRqANguaUiwhbMbm7m7JLU6oLhKew?e=SPSjMf.
